# Central Role of Gimap5 in Maintaining Peripheral Tolerance and T Cell Homeostasis in the Gut

**DOI:** 10.1155/2015/436017

**Published:** 2015-04-07

**Authors:** Mehari Endale, H. Ibrahim Aksoylar, Kasper Hoebe

**Affiliations:** ^1^Department of Molecular and Cellular Immunology, Cincinnati Children's Hospital Research Foundation, MLC7021, Room S5.421, 3333 Burnet Avenue, Cincinnati, OH 45229-3039, USA; ^2^Department of Genetics and Complex Diseases, Harvard School of Public Health, Boston, MA 02115, USA

## Abstract

Inflammatory bowel disease (IBD) including Crohn's disease and ulcerative colitis is often precipitated by an abnormal immune response to microbiota due to host genetic aberrancies. Recent studies highlight the importance of the host genome and microflora interactions in the pathogenesis of mucosal inflammation including IBD. Specifically, genome-wide (GWAS) and also next-generation sequencing (NGS)—including whole exome or genome sequencing—have uncovered a large number of susceptibility loci that predispose to autoimmune diseases and/or the two phenotypes of IBD. In addition, the generation of “IBD-prone” animal models using both reverse and forward genetic approaches has not only helped confirm the identification of susceptibility loci but also shed critical insight into the underlying molecular and cellular pathways that drive colitis development. In this review, we summarize recent findings derived from studies involving a novel early-onset model of colitis as it develops in GTPase of immunity-associated protein 5- (*Gimap5-*) deficient mice. In humans, *GIMAP5* has been associated with autoimmune diseases although its function is poorly defined. Here, we discuss how defects in Gimap5 function impair immunological tolerance and lymphocyte survival and ultimately drive the development of CD4^+^ T cell-mediated early-onset colitis.

## 1. Introduction

The gastrointestinal tract is endowed with a complex immune network that has a major interface with the external environment and thus presents a site with a significant immunological challenge to maintain homeostasis. The maintenance of immune tolerance and gut homeostasis is achieved by an integrated regulation of innate and adaptive immunity but also involves the microbiome itself. The dysregulation of one of these biological components or a combination thereof often precipitates intestinal inflammation or IBD. In general, IBD encompasses two major chronic relapsing inflammatory conditions in the gastrointestinal tract: ulcerative colitis (UC) and Crohn's disease (CD). UC typically involves bloody diarrhea and inflammation involving the rectum that is often extended towards the proximal colon. Infiltration of inflammatory cells is chronic and restricted to the superficial layers of the colonic mucosa. On the other hand, CD is more pleomorphic and is characterized pathologically by discontinuous segments of transmural inflammation that can affect all parts of the GI tract, most commonly the ileocecal region. CD is often presented with development of fistulae and/or strictures while histological granulomata are a key feature. Importantly, the etiology or how dysregulation of the biological components required for gut homeostasis contributes to UC and CD remains poorly defined. An in-depth understanding in the development and/or causes of IBD will require a critical understanding of the interplay between several factors, including genetic susceptibility loci, the host immune system function, the development and composition of the intestinal microflora, and environmental factors such as diet, antibiotic treatment, appendectomy, and hygiene status [1–3].

Recent technical advances that allow for whole genome/exome sequencing [4, 5] and large scale genome wide association studies (GWAS) [6, 7] have led to a dramatic expansion of genetic studies and significantly advanced our understanding of the importance of susceptibility loci associated with chronic (auto-)immune diseases including IBD [4–9]. Not only have NGS approaches been used to identify new and rare variants causing IBD using whole genome and/or whole exome sequencing, but also they have been used to facilitate transcriptome profiling in tissues from IBD patients (RNAseq analysis) and perform epigenomic characterization using CHIP-seq technology. In addition, next-generation sequencing allows for an in-depth analysis of the intestinal microbiome through 16S rRNA sequencing and thus promises to identify the role of microflora in IBD development. To date, more than 160 IBD genes and/or loci have been identified by GWAS [10, 11], most of them contributing modestly (relative risk of <2-fold) to disease susceptibility [12]. The identified loci predominantly represent polymorphisms in genes involved in the innate and/or adaptive immune function [13–15] but also involve genes required for autophagy [16, 17], epithelial barrier function [18], and/or activation of the endoplasmic reticulum stress response [19], indicating the diverse etiology of IBD [13, 20, 21]. The biological consequences and establishment of causality for associated variants still remain a challenging endeavor that relies on in-depth prior knowledge of gene function [22, 23]. As a consequence, for a large number of IBD loci, the functional alleles have not been confirmed and often the causal gene itself is unclear. Thus, the identification of causative genes and alleles remains a significant challenge. Nonetheless, traits that currently have been confirmed as susceptibility genes for IBD and are subject of intense research efforts include* NOD2* [20],* HLA class II* [24],* IL23R* [14], and genes involved in autophagy (e.g.,* Leucine-rich repeat kinase 2* [*LRRK2*] [25],* ATG16L1* [16], and* immunity related guanosine triphosphate M* [*IRGM*]) [17]. For some, gene function is well defined [26, 27]; however, the functional implications of gene variants and how they predispose to colitis often remain elusive [8, 28, 29]. Whereas CD and UC behave as polygenic traits, rare cases of early-onset severe IBD presenting in infancy mostly behave as Mendelian disorders resulting from autosomal recessive mutations in single genes [30–34]. Mutations in* IL10RA*,* IL10RB *[35], or* X-linked inhibitor of apoptosis *(*XIAP*) [36] that cause severe forms of CD in infants born to consanguineous parents are prime examples [37]. Unfortunately, because of the disease severity often seen in early-onset IBD and the low frequency of patients carrying (unique) variants that may be life-threatening, identification of the genetic cause has often proved to be challenging. Current strategies involve resequencing of candidate genes and/or sequencing the whole genome/exome of individual patients by next-generation sequencing. While NGS has the potential to unveil all genome-/exome-wide variants, the understanding of the biological consequences of such variants again is challenging and requires a priori knowledge of gene function [22].

The use of (genetic) animal models has been helpful in providing biological insights into how genetic susceptibility loci affect gut homeostasis and, for instance, has revealed critical immunological pathways that are required for immunological tolerance in the gut [22, 38, 39]. Moreover, such models have revealed insight into the intricate balance between (altered) immune function and the role of microflora to IBD development [40]. To this extent, both forward and reverse genetic approaches have been valuable tools to improve our understanding of genes function, their regulation, and other complex interactions at the cellular and organismal level [22].

Our laboratory has applied an N-ethyl-N-nitrosourea (ENU) mutagenesis approach to identify genes with nonredundant function in lymphocyte development, priming, or effector function. As a result, we have identified a number of germ-line mutants that exhibit impaired peripheral tolerance, lymphocyte survival, and/or T cell activation [22, 41–43]. Among these, an ENU germline, designated* sphinx*, exhibited reduced peripheral T cell survival while developing spontaneous early-onset colitis development. The development of IBD-like intestinal inflammation in Gimap5-deficient mice exhibits hallmark features of IBD development in humans that include (1) a critical role for microbial flora; (2) colitis that is CD4^+^ T cell driven; and (3) a concomitant loss of immunological tolerance, exemplified by a progressive decline in regulatory T cells (T_reg_) numbers and function. Here, we discuss these critical aspects in the context of human IBD and consider the mechanistic pathways by which loss of Gimap5 leads to a loss of immunological tolerance ultimately causing the development of early-onset and severe colitis.

## 2. Gut Homeostasis, Immune Tolerance, and the Microbiome in IBD Development

The intestine represents a potential gateway for microbial pathogens but also contains commensal flora and dietary antigens that require strict immune tolerance. It is therefore no surprise that the gut constitutes the largest lymphoid organ in the body containing an extensive network of secondary lymphoid organs, with an enormous number of leukocytes, including several lymphocyte subpopulations that are uniquely observed in the gut [44, 45]. Upon activation, the intestinal immune system can mount a range of immune effector functions that have the potential to damage host tissue and reduce epithelial barrier function. Thus, a failure to maintain immunological tolerance against commensal flora often results in chronic intestinal inflammation [35].

The intestinal microbiota profoundly affects the immune system development under healthy conditions and thus represents an important environmental determinant of IBD development [46]. This is supported by evidence derived from human studies and studies using mouse models, as reviewed elsewhere [9, 47, 48]. For instance, (genetic) mouse models of intestinal inflammation generally do not develop disease when housed under germ-free conditions [49]. Moreover, T cell-mediated colitis is largely driven by bacterial antigens and fails to develop following nonspecific activation of host T cells. For example, transfer of OVA-specific CD4^+^ T cells from RAG-2^−/−^ OT-II transgenic mice into RAG-2^−/−^ recipients developed colitis only when recipient mice were colonized with OVA-expressing* Escherichia coli*, not with control* Escherichia coli* [50, 51]. This finding has led to a particular focus in understanding the role of intestinal microbiota, that is, its composition, regulation, and interaction with the host immune system, in the development of IBD. The gastrointestinal tract harbors more than 10^14^ microorganisms of ~1000 species [52, 53], mostly contained within the colon [54]. Over 90% of these consist of Bacteroidetes (gram negative) and Firmicutes (gram positive) bacteria. Specific* Bacteroides* species directly regulate antimicrobial peptide expression by intestinal epithelium through activation of Toll-like receptors (TLR) expressed on Paneth cells [55]. Moreover, the presence of specific bacterial species shapes adaptive immune functions within the intestines, including Enterobacteriaceae and Bacteroidaceae [56] for TCR*αβ* intraepithelial lymphocytes;* Bacteroides fragilis* [57] and a mixture of Clostridia strains [58] for T regulatory cells; and* cytophaga-flavobacterium-bacteroidetes* and segmented filamentous bacterium for Th17 cells [59–61]. Thus, changes in the composition of commensal microbiota-(dysbiosis) may present a critical determinant of host immune responses and thereby contribute to the development of IBD [29]. Interestingly, studies involving 16S rRNA sequencing from gut biopsy or stool samples revealed a detectable difference between the intestinal microbiota in the two forms of IBD (CD and UC) compared to healthy controls [62]. However, whether the observed dysbiosis in microbiota is directly associated with the presence of IBD susceptibility loci or a consequence of intestinal inflammation* per se* is currently unclear and an area of intense inquiry. A prime example of bacterial species driving colitis is provided by studies involving* Helicobacter hepaticus*—a commensal bacterium with opportunistic pathogenic potential [57, 63]. Although colonization of wild-type C57BL/6J mice with* H. hepaticus* does not result in inflammation or disease,* H. hepaticus* induces colitis in IL10^−/−^ [64] or SCID/Rag2^−/−^ hosts that received naïve CD4^+^CD45RB^high^ T cells [63]. This colitis model is driven by homeostatic proliferation of naïve T cells through bacterial antigens including the flagellar antigen of* H. hepaticus* [65]. Colitis induction in this model is only observed in the absence of T_reg_ cells allowing for robust CD4^+^ T cell effector responses. Overall, these observations suggest that perturbations in gut microbiota and host immune system underlie the development of intestinal inflammation and IBD—an etiology referred to as the two-hit hypothesis [66].

## 3. Monogenic Causes of IBD

Interestingly, a large number of IBD susceptibility loci identified by GWAS studies are shared with other complex (auto-)immune diseases such as type-1 diabetes, celiac disease, multiple sclerosis, and systemic lupus erythematosus [10]. This is primarily due to the fact that these loci represent genes involved in immune cell signaling, including T cell differentiation, immune tolerance, and/or innate immune responses [28, 67, 68]—immunological pathways that are critical determinants for (auto-)immune disease. Clear examples of such loci are loss-of-function mutations in either IL10RA or IL10RB [35]. These mutations are linked with severe, early-onset enterocolitis in children—a pathology that is also observed in mice lacking either* Il10* [69, 70] or* Il10rb* [31, 35]. Changes in* Il-10r* variants are functionally linked to alterations in hematopoietic cell function and colitis can generally be cured through hematopoietic stem cell transplantation [71].

Interleukin-10 (IL-10) is a pleiotropic cytokine with a multitude of anti-inflammatory and immunoregulatory functions, which is secreted by a variety of cell types and is critical for maintaining immune homeostasis of the gut [72, 73]. For instance, IL-10 modulates the function of APCs through inhibiting phagocytosis, downregulating the expression of MHCs and costimulatory molecules, and decreasing the production of proinflammatory cytokines and chemokines in IBD [74]. Moreover, IL-10 directly restricts the differentiation of Th cells [70, 74] and maintains the suppressive activity of T_reg_ cells [75]. Consistent with this, T cell-specific [76] or FoxP3^+^  T_reg_-specific [77] deletion of* Il10* results in spontaneous colitis, highlighting the importance of T_reg_-derived IL-10 in preventing intestinal inflammation. On the other hand, a recent study suggests that macrophages are a prime cell target for IL-10 activity in the gut in that loss of* Il-10ra* specifically on macrophages resulted in spontaneous colitis development [78]. Overall, these studies establish IL-10 as a central mediator in gut homeostasis affecting both innate and adaptive immune responses.

## 4. The Role of CD4^+^ T Cells in Colitis

The key challenge of the intestinal immune system is to properly respond to pathogens while maintaining immune tolerance towards commensal bacteria and food antigens [79]—a process that requires complex cellular and molecular regulatory mechanisms [45, 80]. Particularly, the presence of unique immunosuppressive CD4^+^ T cell populations has been described in the intestine that control immune homeostasis and prevent inflammation towards harmless foreign antigens [81]. Importantly, increased accumulation of CD4^+^ T cells in the intestine is a key feature of inflammatory bowel disease [9, 82] and presents an important therapeutic target. Intestinal CD4^+^ T cell populations can be broadly classified based on function into effector CD4^+^ T cells and regulatory CD4^+^ T cells.

Effector CD4^+^ T cells, also referred to as helper T (Th) cells, play a critical role in the execution of immune functions. These include the development of antigen-specific CD8^+^ T and B cell responses and inflammatory cytokine production causing the recruitment of effector cells such as neutrophils. Whereas early studies primarily focused on the functional distinction between Th1 (or IFN-*γ* ^+^ producing CD4^+^ T cells) and Th2 cells (interleukin 4-producing T cells), more in-depth studies in mice suggested that Th2 cells were largely absent in healthy mouse colonies in the absence of intestinal parasites [83]. Importantly, a third subset, the Th17 subset of CD4^+^ T cells, has recently been described as the major T cell population within both healthy and inflamed intestinal mucosa [84]. The identification of this subset has almost entirely shifted the focus on this cell type as a driver of disease in both experimental models and human IBD. Th17 cells produce a large number of cytokines, including IL-17A and IL-17F—key cytokines involved in the recruitment and activation of granulocytes and critical to the host response against extracellular bacteria. Importantly, microbiota-specific memory Th17 cells are far more potent in inducing colitis in recipient mice compared to Th1 cells [85]. Moreover, a correlation between IL17 levels and disease severity in human IBD patients has been observed [86] suggesting a key role for Th17 cells and cytokines in IBD. Although the classification of these T helper cells suggests a specific and unique cytokine production profile, the CD4^+^ T cells isolated from lamina propria undergoing active colitis can express both IL-17 and IFN*γ*, indicating the unique plasticity of Th17 cells and their ability to convert into Th1 cells [87]. Given that Th17 cells are the main CD4^+^ T cell population in the intestinal tract, this plasticity is thought to be of critical importance to adapt to changes in the local intestinal environment and mount a proper immune response while maintaining gut homeostasis.

The importance and dominance of regulatory T cells and their immunosuppressive function are demonstrated by the fact that the majority of individuals do not develop gut inflammation despite an enormous microbial and antigenic load within the intestine. Moreover, transfer of naïve CD4^+^CD45RB^high^ CD4^+^ T cells in lymphopenic hosts such as Rag1/2^−/−^ or SCID mice induces lymphopenia-induced T cell activation and colitis only in the absence regulatory T cells (reviewed in [45, 88]).

Thus, T_regs_ cells play a critical role in maintaining immune homeostasis and limiting autoimmune responses by modulating cells of both the innate and the adaptive immune systems. The main types of regulatory cells in the gut are the natural (thymic) and adaptive (induced) CD4^+^FoxP3^+^  T_regs_, as well as Tr1 and Th3 cells [89]. The effector pathways by which T_regs_ induce tolerance are multiple and include secretion of inhibitory cytokines such as IL-10 and transforming growth factor-*β* (TGF-*β*), granzyme-mediated cytolysis of target cells, expression of cytotoxic T-lymphocyte antigen- (CTLA-) 4 resulting in T cell inhibition, and metabolic disruption [45, 90, 91]. Impaired immune regulation by T_reg_ cells will result in a loss of immunological tolerance in the gut and cause colitis. Such deficiencies may stem from inadequate numbers of T_reg_ cells—due to impaired development, proliferation, or survival—or defects in immunosuppressive function intrinsic to T_reg_ cells. Alternatively, pathogenic effector T cells may be resistant to suppression by T_reg_ cells. At the site of inflammation, effector T cells are reported to develop mechanisms of resistance to T_reg_ regulation [92, 93], although the underlying mechanisms remain poorly defined.

In humans, the critical role for T_reg_ cells in preventing gut inflammation is further supported by the finding that individuals with genetic abberations in IPEX causing functional impairment of the transcription factor FoxP3 develop severe bowel inflammation [94]. Moreover, patients with genetic mutations in FoxP3 who lack or have nonfunctional T_regs_ exhibit severe intestinal inflammation associated with lymphocytic infiltration of the intestinal mucosa [95, 96]. Similarly, mice lacking FoxP3^+^  T_regs_ [92, 97] or lacking the ability to suppress via T_reg_-derived cytokines such as IL-10 [45, 89], IL-35 [98], and TGF*β* [99] develop severe colitis. Together, these studies highlight the importance of CD4^+^ T cells, particularly T_reg_ cells, in maintaining gut homeostasis. In addition, they point to monogenic causes of IBD that specifically affect T_reg_ function ultimately leading to loss of immunological tolerance and gut inflammation.

## 5. GIMAP5: A Critical Determinant of T Cell Survival and Peripheral Tolerance

**Figure 1:**
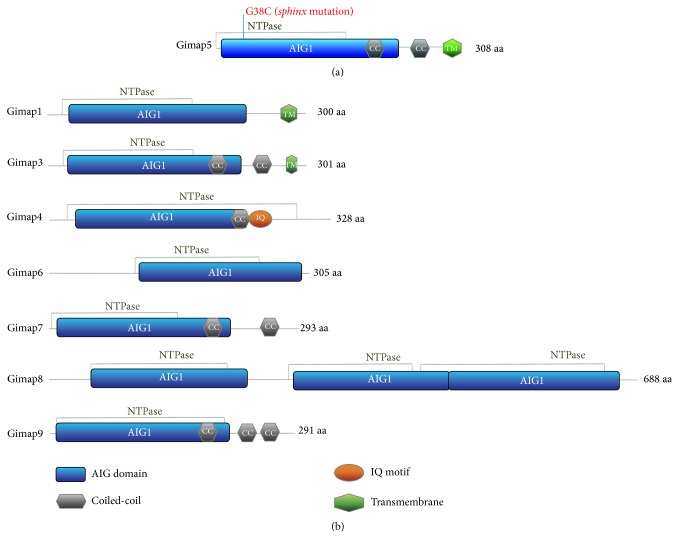
Predicted structural domains within mouse Gimap5. (a) Mouse Gimap5 is a 308-amino acid protein that contains an AIG1 domain (residues 24–227), a P-loop NTPase domain (residues 1–168), two coiled-coil domains (residues 187–221 and 239–265), and a transmembrane domain (residues 284–304). The G→C missense mutation in* sphinx* mice at residue 38 is indicated. (b) Schematic overview of the domain features present in the different Gimap family members.

Recently, studies have identified the GTPase of immunity-associated protein 5 (GIMAP5) as a key factor in maintaining T cell homeostasis and immunological tolerance. GIMAP5 is part of the family of GIMAP proteins, which are predominantly expressed in lymphocytes and regulate lymphocyte survival during development, selection, and homeostasis [100–106]. Members of this family share a GTP-binding AIG1 (avrRPT2-induced gene-1) domain, derived from an AIG1 resistant gene first described in* Arabidopsis thaliana* that was induced upon infection with* Pseudomonas syringae type III*. The AIG domain is conserved across vertebrates and angiosperms and, in vertebrates, the family consists of seven (human and rat) and eight (mouse) members that are clustered within a tight single region on chromosomes 7, 4, and 6, respectively, ([107–110] and (Figure 1)). Mouse Gimap5 is a 308-amino acid protein that contains an AIG1 domain (residues 24–227) comprising five GTP-binding motifs (G1–G5), a P-loop NTPase domain (residues 1–168), two coiled-coil domains (residues 187–221 and 239–265), and a transmembrane domain (residues 284–304) ([105, 106] and (Figure 1)). Recent crystallographic studies revealed that the Gimap proteins manifest a nucleotide coordination and dimerization mode similar to dynamin GTPase—a component essential for the scission and fusion of cellular vesicular compartments such as endosomes [111, 112]. Members of the Gimap family appear to be expressed in different subcellular compartments, with Gimap5 localizing in multivesicular bodies (MVBs) and lysosomes in lymphocytes [113]. Their function in lymphocytes, however, remains poorly defined.

Genetic aberrancies of* GIMAP5* have been linked to impaired immunological tolerance, lymphocyte survival, homeostasis, and autoimmunity in a variety of species including humans, mice, and rats. In humans, polyadenylation polymorphisms in* GIMAP5* are associated with increased concentrations of IA2 autoantibodies in type 1 diabetes (T1D) patients [114] and an increased risk of systemic lupus erythematosus SLE [115, 116]. Moreover, in patients with T1D, expression of several GIMAP genes including GIMAP5 is reduced in T_reg_ cells compared to healthy individuals [117]. In a spontaneous rat model of type I diabetes (the BioBreeding diabetic prone (BB-DP) rats), abnormal thymocyte development and premature death of peripheral CD4^+^ and CD8^+^ T-cells [110, 118, 119] were linked to a frame shift mutation in GIMAP5, designated* lyp*, causing a truncated nonfunctional protein (*GIMAP5* ^*lyp/lyp*^) [100–106]. In the presence of the diabetogenic MHC locus IDDM1, this* lyp* mutation is essential for diabetes onset in BB-DP rats ultimately triggering lethal disease [105, 106, 110]. A similar loss of lymphocyte survival is observed in* Gimap5* ^−/−^ null mice [120]. However, the loss of lymphocyte survival in* Gimap5* ^−/−^ mice is not limited to T cells, but also extends to reduced survival of NK, iNKT, and B cells with extensive extramedullary hematopoiesis observed in the liver [120].

These observations were confirmed by an N-ethyl-N-nitrosourea (ENU) induced* Gimap5*-germline mutant identified in our laboratory—designated* sphinx. *ENU is a widely used mutagen to create random germline point mutations in mice and has proven to be an effective approach to probe and identify critical genes for any phenotype of interest, for example, colitis or development/function of the immune system [22, 121]. Phenotypes causing ENU mutations primarily involve missense mutations (~61%) or nonsense mutations (10%) (source: http://mutagenetix.utsouthwestern.edu/), the type of genetic variants that can be found in humans. The* sphinx* mutation involved a G→T point mutation in* Gimap5* resulting in a G38C substitution in the predicted GTP-binding domain of Gimap5 [42]. The mutation destabilized the protein and caused a complete loss-of-function similar to the published Gimap5 KO [26]. Specifically, the* sphinx* mutant exhibited a similar reduced lymphocyte survival, including loss of NK cells, CD4^+^ T, CD8^+^ T, and B cells to the* Gimap5* knockout mice reported. The causative germline mutation involved a single G→T point mutation in Gimap5. This mutation resulted in a G38C substitution in the predicted GTP-binding domain of Gimap5, destabilizing the protein and causing a complete loss-of-function. Interestingly, from birth until weaning,* sphinx* (or* Gimap5* ^*sph/sph*^) mice appear outwardly healthy. However, after 7-8 weeks of age, mice lose weight and develop severe colitis, exemplified by goblet cell depletion, lamina propria leukocyte infiltration, epithelial cell hyperplasia, and crypt loss [42, 43]. The severe colitis likely contributed to the early mortality of* Gimap5* ^*sph/sph*^ mice, which generally occurred by 14 weeks of age. Interestingly, antibiotic treatment blocked intestinal inflammation in* Gimap5* ^*sph/sph*^ mice, suggesting a critical role for the microbiome also in this spontaneous model of colitis. Overall, inflammation of the gut in* Gimap5* ^*sph/sph*^ mice is early-onset and behaves as a monogenic trait, thus very similar to mutations in IL10RA, IL10RB, or XIAP [32, 33, 122, 123].

## 6. *Gimap5* ^*sph/sph*^ Mice: A Novel T Cell-Mediated Colitis Model

**Figure 2:**
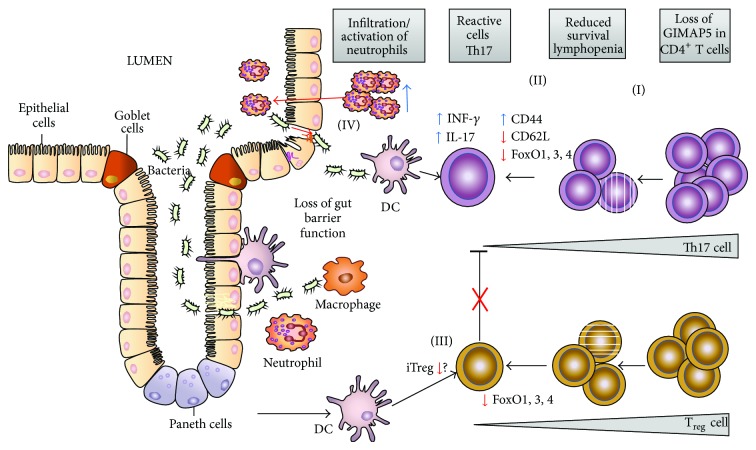
Schematic representation of the key events causing colitis in* Gimap5*-deficient mice. Loss of Gimap5 leads to reduced survival of lymphocytes (I) including CD4^+^ T cells with remaining T cells exhibiting a characteristic LIP phenotype (CD44^high^; CD62L^low^) and polarization towards Th17 (II). Importantly, during the onset of CD4^+^ T cell lymphopenia, a progressive loss of full-length FoxO1, FoxO3, and FoxO4 expression is observed that correlates with a loss of T_reg_ induction (iTreg) and function in the gut tissue (III). The lack of T_reg_ immunosuppressive activity (indicated by the red X) triggers activation of CD4^+^ Th1/Th17 cells in the gut causing production of IL17 and IFN*γ* cytokines and subsequent infiltration of macrophages/neutrophils that further amplify intestinal inflammation and a loss of epithelial barrier function (IV) and may ultimately lead to neutrophil transepithelial migration (for an extensive review on neutrophils in IBD pathogenesis, see [133]).

*Gimap5* ^*sph/sph*^ mice exhibit an absence of NK or CD8^+^ T cell populations in peripheral lymphoid organs. Interestingly, relatively normal thymocyte development occurs, including CD4^+^ T cell, CD8^+^ T cell, and Foxp3^+^ regulatory T cell lineages [42]. Nonetheless,* Gimap5* ^*sph/sph*^ mice exhibit a progressive reduction in circulating CD4^+^ T cells and the CD4^+^ T cells that remain after five weeks of age exhibit a lymphopenia-induced proliferation (LIP) phenotype (CD44^high^ and CD62L^low^), a T cell phenotype associated with autoimmunity [124]. Interestingly, despite their reduced survival,* Gimap5* ^*sph/sph*^ CD4^+^ T cells produced exceeding amounts of IFN*γ* and IL-17A compared to wild-type CD4^+^ T cells and exhibited spontaneous activation in the* Gimap5* ^*sph/sph*^ gut tissue pointing to a potentially critical role of CD4^+^ T cells in this disease model. Indeed, antibody-mediated CD4-depletion* in vivo* prevented colitis in these mice corroborating the importance of CD4^+^ T cells in the pathogenesis. The lymphopenia and expression of CD44^high^CD62L^low^ markers by CD4^+^ T cells (Figure 2) are indicative of lymphopenia-induced proliferation and resemble the CD4^+^ T cell phenotype first described in the adoptive transfer T cell model of colitis [125]. As mentioned, the development of CD4^+^ T cell-induced colitis in* Gimap5* ^*sph/sph*^ can be prevented by antibiotic-treatment, again confirming the critical role of the microbiota in T cell activation [43]. Although the intestinal microbiota provide a potentially large source of foreign antigens that may drive the T cell response towards gut tissue, it is important to note that many autoimmune diseases are associated with immune-deficiencies which result in lymphopenia and subsequent “homeostatic” proliferation. The genetic and molecular basis of how these complex processes are controlled still remains incompletely defined. T_reg_ cells have been implicated as a critical factor in the development of disease following homeostatic proliferation and a similar critical role for T_reg_ cells was observed in the colitis development in* Gimap5* ^*sph/sph*^ mice. Specifically,* Gimap5* ^*sph/sph*^ mice fail to maintain a T_reg_ population with immunosuppressive function. Whereas relatively normal numbers of Foxp3^+^  T_reg_ cells were found in spleen and LNs of 3-week-old mice, T_reg_ cell numbers were significantly reduced in 6-week old mice [43]. More importantly, a progressive loss of T_reg_ function in MLN of* Gimap5* ^*sph/sph*^ mice was observed. Whereas T_reg_ cells from 4-week-old* Gimap5* ^*sph/sph*^ mice showed a slight but significant reduction in their ability to suppress wild-type CD8^+^ T cell proliferation* in vitro*, T_reg_ cells from older (6-week-old)* Gimap5* ^*sph/sph*^ mice were incapable of suppressing wild-type CD8^+^ T cell proliferation, suggesting a critical loss of T_reg_ function and survival to be responsible for colitis development in these mice (Figure 2). Indeed, transfer of wild-type CD4^+^CD25^+^  T_reg_ cells into* Gimap5* ^*sph/sph*^ early on prolonged survival, prevented increased CD4^+^ T cell effector function in the MLN, and protected these mice from colitis [43]. Together, these data indicate that Gimap5 is a critical determinant of T_reg_ survival and function, thereby controlling gut homeostasis. The critical role of Gimap5 in T_reg_ survival/function is also evident in Type 1 diabetes in BioBreeding rats [105, 106] and may clarify why polyadenylation polymorphisms in* GIMAP5*, leading to rather subtle changes in gene expression, are associated with human autoimmune diseases such as T1D [114] and SLE [115].

## 7. Molecular Determinants of Peripheral Tolerance in the Absence of Gimap5 

Given the loss of T_reg_ development/function, key questions currently center on understanding the molecular pathways by which Gimap5 controls T cell survival and peripheral tolerance. A number of studies have implicated Gimap5 to interact with Bcl2 members in mitochondria and implicated a critical role for Gimap5 in controlling proapoptotic pathways in T cells. Data in our laboratory, however, revealed no improved survival of lymphocytes (or prevention of colitis for that matter) when* Gimap5* ^*sph/sph*^ mice were crossed to* Bim*-deficient or* Bax/Bak*-deficient backgrounds (Aksoylar and Hoebe; unpublished data) suggesting that the reduced T cell survival is likely independent of the classical proapoptotic pathways. In terms of peripheral tolerance, a striking similarity is observed with the phenotypes reported in mice deficient in the family of Fork-head box group O (Foxo) transcription factors. The family of Foxo transcription factors contains 4 members of which three (Foxo1, Foxo3, and Foxo4) have overlapping patterns of expression and transcriptional activities and they play an essential role in the quiescence and survival of CD4^+^ T cells [126, 127]. In addition, Foxo expression has been reported to be essential for T_reg_ cell development and function [128, 129]. The potential mechanisms by which Foxo transcription factors control T_reg_ development and function have been described in detail and include their role as coactivators downstream of the TGF*β* signaling pathway by (1) interacting with SMAD proteins [130, 131] and by (2) directly regulating the induction of a number of T_reg_ cell associated genes, including Foxp3 itself but also CTLA-4 and CD25 [128, 129]. Importantly, CD4^+^ T cells from* Gimap5* ^*sph/sph*^ mice revealed a complete absence of Foxo1, -3a, and -4 proteins. This effect was predominantly observed at the protein level with relatively normal RNA levels in CD4^+^ T cells, suggesting that regulation of Foxo3 and Foxo4 protein expression occurs predominantly at the posttranslational level. Interestingly, the loss of Foxo expression was progressive and correlated with the loss of immunological tolerance in Gimap5-deficient mice. Importantly, the loss of Foxo expression in* Gimap5* ^*sph/sph*^ CD4^+^ T cells was specifically observed in cells undergoing LIP, which may suggest degradation of Foxo expression due to constitutive homeostatic activation of T cells (Figure 2). Although T cell activation in general results in a brief transient loss of Foxo expression [132], loss of Foxo expression is not observed following transfer of wild type CD4^+^ T cells into lymphopenic Rag2-deficient hosts (Aksoylar, Hoebe; unpublished results), suggesting that the loss of Foxo proteins in Gimap5-deficient CD4^+^ T cells involves a unique degradation mechanism. Importantly, the loss of Foxo expression in* Gimap5* ^*sph/sph*^ CD4^+^ T cells correlated with a loss of T_reg_ population and function and likely represents an important determinant of the colitis pathology observed in these mice.

## 8. Conclusion

A genetic alteration in Gimap5 has been strongly linked with reduced T cell survival and loss of immunological tolerance in both animal models and human studies. This results in predisposition to a variety of autoimmune related diseases including T1D, SLE, and colitis. Despite the profound impact of Gimap5 deficiency in terms of both lymphoid survival and peripheral tolerance, very little is understood about the molecular mechanisms underlying these robust phenotypes. Thus, a number of critical questions remain to be addressed that include (i) what is the molecular function of Gimap5 in T cells following activation?, (ii) what are the mechanistic pathways by which loss of Gimap5 causes reduced lymphocyte survival and peripheral tolerance* in vivo*?, and (iii) why do CD4^+^ T cells in Gimap5-deficient mice exhibit loss of Foxo expression at the posttranslational level?

Finally, given the severe phenotypes related to the host immune system observed in both mouse and rat Gimap5-deficient models, a* GIMAP5* null phenotype in humans is expected to result in a severe immunodeficiency, although the phenotype has yet to be described. Such a severe immunodeficiency would be predicted to present in infancy as a monogenic trait and, with the current sequencing capacity and efforts,* de novo* mutations in* GIMAP5* should be considered prime causal candidates. Regardless, the detailed mechanistic insight into the loss of T cell survival and immunological tolerance in* Gimap5* ^*sph/sph*^ mice may ultimately help our understanding as to how polyadenylation polymorphisms in GIMAP5 predispose to T1D or SLE in humans. In addition, these studies point to a new candidate genetic susceptibility locus that should be taken into consideration for variants identified in early-onset colitis in pediatric patients.
